# Outcome and response to different management regimens in pediatric patients with immune thrombocytopenia (ITP)

**DOI:** 10.1007/s00277-025-06626-1

**Published:** 2025-09-26

**Authors:** Rasha AbdelRaouf AbdelAziz, Dalia El-Sayed, Fatma El Zahraa Ahmed, Mohammed Al Komy

**Affiliations:** Department of Pediatrics, Pediatric Hematology and BMT unit, Faculty of Medicine, Cairo University, Cairo, Egypt

**Keywords:** Immune thrombocytopenia (ITP, Corticosteroids, Thrombopoietin receptors agonists (TPO-RA), Intravenous immunoglobulin (IVIG).

## Abstract

Immune thrombocytopenia (ITP) is the most common acquired bleeding disorder in children and a frequent source of clinical concern. This study aimed to evaluate the clinical outcomes and treatment responses to different therapeutic lines. This observational study included 90 children with newly diagnosed ITP who were registered and followed at Cairo University Children’s Hospital between June 2022 and December 2023. The study cohort consisted of 40 males (44.4%) and 50 females (55.6%), with a mean age of 5.3 years. The mean platelet count at presentation was 9.9 ± 11.9 × 10⁹/L, increasing to 384.6 ± 141.0 × 10⁹/L at six months post-treatment. Six patients (5.8%) experienced spontaneous recovery without treatment, while 84 patients (94.2%) received therapeutic interventions including corticosteroids or intravenous immunoglobulin (IVIG). By three months, 61 patients (68.9%) had responded to treatment, while 29 patients (32.2%) required second-line therapy with thrombopoietin receptor agonists (TPO-RAs). Corticosteroids remain the cornerstone of first-line therapy in newly diagnosed pediatric ITP. Patients who do not respond to initial treatment or experience relapse demonstrate favorable outcomes with TPO-RA therapy.

## Introduction

Childhood primary immune thrombocytopenia is the most common acquired autoimmune bleeding disorder, characterized by isolated thrombocytopenia (peripheral blood platelet count less than 100 × 10⁹/L) in the absence of other causes or disorders that may induce thrombocytopenia [1].

ITP is classified into primary and secondary forms depending on the cause, based on the American Society of Hematology’s (ASH) 2019 recommendations. Antibodies against reticuloendothelial system macrophages and platelet surface antigens are the cause, leading to defects in thrombopoiesis and platelet destruction [2].

Clinically, ITP is often a self-limited condition, resolving spontaneously in approximately 85% of cases within six months without the need for treatment [3]. However, a significant subset of patients requires medical intervention and may experience a chronic course. Based on duration, ITP is classified as newly diagnosed (diagnosis to 3 months), persistent (3 to 12 months), and chronic (lasting more than 12 months) [4].

Management of ITP remains a subject of debate, with the primary goal being to maintain a safe hemostatic platelet level to prevent life-threatening bleeding complications such as intracranial hemorrhage—although rare, this remains a critical concern [5]. Various treatment options exist. A “watch and wait” or observation strategy is recommended for pediatric patients with no or minor bleeding symptoms. Corticosteroids remain the most commonly used first-line therapy for children with clinically significant bleeding, those at risk of bleeding, or those experiencing diminished health-related quality of life [6]. According to American Society of Hematology (ASH) latest guidelines; short course of steroids < 6 weeks is recommended [6]. Intravenous immunoglobulin G (IVIG) or anti-D immunoglobulin are alternatives for patients in whom corticosteroids are contraindicated or otherwise not preferred [7].

Second-line therapies are reserved for children with ITP persisting for three months or longer who remain at risk of bleeding and do not respond to first-line treatment [7].

The current study aimed to compare the efficacy of different treatment options for pediatric acute and persistent ITP, focusing on patient response, treatment safety, and overall disease outcome.

## Methods

This observational study retrospectively reviewed data from pediatric patients aged 1 to 14 years diagnosed with immune thrombocytopenia (ITP) who presented to the Pediatric Hematology Department at Cairo University Children’s Hospital between June 2022 and December 2023. Patient files were thoroughly reviewed, and relevant data were extracted for analysis. Patients with secondary thrombocytopenia, bone marrow failure, or myelodysplastic syndrome were excluded from the study. Informed written consent was obtained from parents or guardians, and assent was obtained from the children when appropriate. All data were handled confidentially, with participant anonymity maintained through the use of serial identification numbers. The study protocol was approved by the Ethical Committee of the Faculty of Medicine, Cairo University **(REC code: MS28-2024).**

Bleeding episodes were assessed at each visit using the ITP-specific bleeding scale based on the ISTH-SSC Bleeding Assessment Tool (BAT) score. The bleeding manifestations were categorized into three groups: skin (S), mucosal (M), and organ (O). The scale varies from 0 to 4 for epistaxis and for organ bleeding, except ocular and intracranial bleeding (grade 0 and 2 to 4). The rest bleeding sites (cutaneous and mucosal) were classified in four grades (0–3). Grade 5 is related to any fatal hemorrhage [8].

Assessments were conducted at presentation and subsequently at 1 week, 1 month, 3 months, and 6 months to evaluate treatment response. Clinical data—including demographics, preceding illnesses, symptoms, and drug therapy—and laboratory data, primarily complete blood counts, were collected at baseline, weekly during the first two weeks, and monthly thereafter for six months. Data were obtained through review of medical records as well as direct interviews with patients and their legal guardians.

The choice of treatment was tailored to each patient, considering clinical features, bleeding risk, lifestyle considerations, and the preferences of both caregivers and treating physicians.

The quality of response to treatment was recorded according to the latest American Society of Hematology (ASH) definitions [7], supplemented by the terminology recommended by the International Working Group:

- **Complete Response (CR)**: Platelet count ≥ 100 × 10⁹/L and absence of bleeding.
- **Response (R)**: Platelet count ≥ 30 × 10⁹/L, with at least a two-fold rise from baseline, and absence of bleeding.
- **No Response (NR)**: Platelet count < 30 × 10⁹/L, or less than a two-fold rise from baseline, or presence of bleeding.
- **Loss of CR or R**: For CR, platelet count falls below 100 × 10⁹/L or bleeding occurs; for R, platelet count falls below 30 × 10⁹/L, less than a two-fold rise from baseline, or bleeding occurs.

### Statistical analysis

The Statistical Package for Social Sciences (IBM-SPSS/PC/VER 24) was used to evaluate the data that had been obtained. In descriptive statistics, qualitative data were presented as frequencies and percentages, while continuous variables were represented as mean ± standard deviation, median, and range. The differences in frequency distribution across the several groups were compared using the chi square, Fisher Exact, and Monte Carlo exact tests. Student t-test and Mann-Whitney U test was calculated to test the mean/median differences in continuous variables between groups. For variables with more than two categories, ANOVA test and Kruskal Wallis test was calculated to test the mean/median differences in continuous variables between groups. Pearson’s/Spearman rank correlation co-efficient was calculated for univariate correlations. When the p-value was less than 0.05, the test findings were deemed significant.

## Results

The study included 90 pediatric patients diagnosed with ITP between June 2022 and December 2023. The cohort comprised 40 males (44.4%) and 50 females (55.6%), yielding a female-to-male ratio of 1.2:1. The mean age of the patients was 5.3 ± 2.9 years, and the mean duration from symptom onset to presentation was 3.2 ± 1.8 days.

Bleeding severity was assessed using the Bleeding Assessment Tool (BAT) score, with the majority of patients (63.3%) presenting with scores ranging from 5 to 7, indicating moderate bleeding symptoms.

Baseline laboratory data for the study population are summarized in Table 1.

**Table 1:** Baseline laboratory data of the study patients (n=90)

Variable	Value (Mean ± SD)
**Hemoglobin (g/dL)**	11.3 ± 1.0
**TLC (x10**^**9**^**/L)**	7.7 ± 2.5
**ANC (/µL)**	2234.8 ± 211.8
**ALC (/µL)**	2732.4 ± 1046.2
**Platelet (x10**^**9**^**/L)**	9.9 ± 11.9
**Reticulocyte %**	1.8 ± 0.5

Abbreviations: TLC (Total leukocyte count), ANC (Absolute neutrophilcount), ALC (Absolute lymphocyte count).

Newly diagnosed ITP patients with no or only minor bleeding were managed conservatively with observation alone. No medical treatment was prescribed, aside from providing family education and guidance on avoiding accidental trauma and medications that could increase bleeding risk.

**Corticosteroids** were administered to children who exhibited clinical bleeding and/or a diminished health-related quality of life. Treatment regimens included:

- **Oral prednisone** at a dose of 2–4 mg/kg/day (maximum 120 mg/day) for 1–2 weeks, followed by tapering over an additional 1–2 weeks.
- **Intravenous (IV) pulse methylprednisolone** (20–30 mg/kg/day) for 3–5 days, followed by oral prednisolone (2–4 mg/kg/day, maximum 120 mg/day) for 1–2 weeks, then tapered over 1–2 weeks.
- **IV pulse methylprednisolone alone** (20–30 mg/kg/day for 3–5 days), not followed by oral steroids.

**Intravenous Immunoglobulin (IVIG)** at a dose of 0.8–1 g/kg/day for 2 consecutive days was used in cases where corticosteroids were contraindicated or not preferred.

**Second-line treatments** were initiated in patients who did not achieve a response or lost response after three months of initial therapy. **Thrombopoietin receptor agonists (TPO-RAs)** were the main agents used, including:

- **Eltrombopag**, starting at 25 or 50 mg/day depending on age, titrated based on platelet response and bleeding symptoms, up to a maximum of 75 mg/day. Median dose was 50 mg/day.
- **Romiplostim**, initiated at 1 mcg/kg subcutaneously (SC) weekly and titrated up to a maximum of 10 mcg/kg SC weekly based on platelet count and bleeding status. Median dose was 5 mcg/kg/day.

The choice of TPO-RA was based on patient and physician preference as well as treatment availability.

An analysis of the first-line treatments administered to the study cohort revealed that 36 patients (40%) received intravenous (IV) methylprednisolone followed by oral prednisolone, 20 patients (22.2%) received oral prednisolone alone, 16 patients (17.8%) were treated with intravenous immunoglobulin (IVIG), 12 patients (13.3%) received IV methylprednisolone alone, and 6 patients (6.7%) were managed with observation only, without a ny pharmacological treatment.

Follow-up of these patients demonstrated clinical improvement in bleeding manifestations, as 46.7% of the cohort achieved a BAT score of less than 2 after three months of treatment, regardless of the treatment modality used. These findings are summarized in Table 2

**Table 2:** Comparison of BAT scores among patients receiving different first line of treatment (n=90)

	Observation (*n*: 6)	Oral prednisolone (*n*: 20)	IV methylpre-dnisolone (*n*: 12)	IV methylprednisolone followed by oral prednisolone (*n*: 36)	IVIG (*n*: 16)	*p*-value
	Number (%)					
Initial BAT score						
**Score less than 2**	0 (0%)	0 (0%)	0 (0%)	0 (0%)	0 (0%)	
**Score 2–5**	4 (66.7%)	9 (45%)	4 (33.3%)	9(25%)	3 (18.8%)	0.282
**Score 5–7**	2 (33.3%)	10 (50%)	8 (66.7%	24 (66.7%)	13 (81.3%)	
**Score 8–10**	0 (0%)	1 (5%)	0 (0%)	3 (8.3%)	0 (0%)	
**BAT score after 1 week**						
**Score** **less than 2**	0 (0%)	0 (0%)	0 (0%)	0 (0%)	0 (0%)	
**Score 2–5**	4 (66.7%)	9 (45%)	4 (33.3%)	9 (25%)	3 (18.8%)	0.206
**Score 5–7**	2 (33.3%)	10 (50%)	8 (66.7%)	27 (75%)	13 (81.3%)	
**Score 8–10**	(0%)	1 (5%)	(0%)	(0%)	(0%)	
**BAT score after 3 months**						
**Score less than 2**	6 (100%)	8 (40%)	6 (50%)	15 (41.7%	7 (43.8%)	0.225
**Score 2–5**	0 (0%)	8 (40%)	2 (16.7%)	15 (41.7%)	6 (37.5%)	
**Score 5–7**	0 (0%)	4 (20%)	4 (33.3%)	6 (16.7%)	3 (18.8%)	
**Score 8–10**	0 (0%)	0 (0%)	0 (0%)	0 (0%)	0 (0%)	
**BAT score after 6 months**						
**Score less than 2**	6(100%)	20(100%)	12(100%)	36(100%)	16(100%)	
**Score 2–5**	0(0%)	0(0%)	0(0%)	0(0%)	0(0%)	
**Score 5–7**	0(0%)	0(0%)	0(0%)	0(0%)	0(0%)	
**Score 8–10**	0(0%)	0(0%)	0(0%)	0(0%)	0(0%)	

**Fig. 1:**
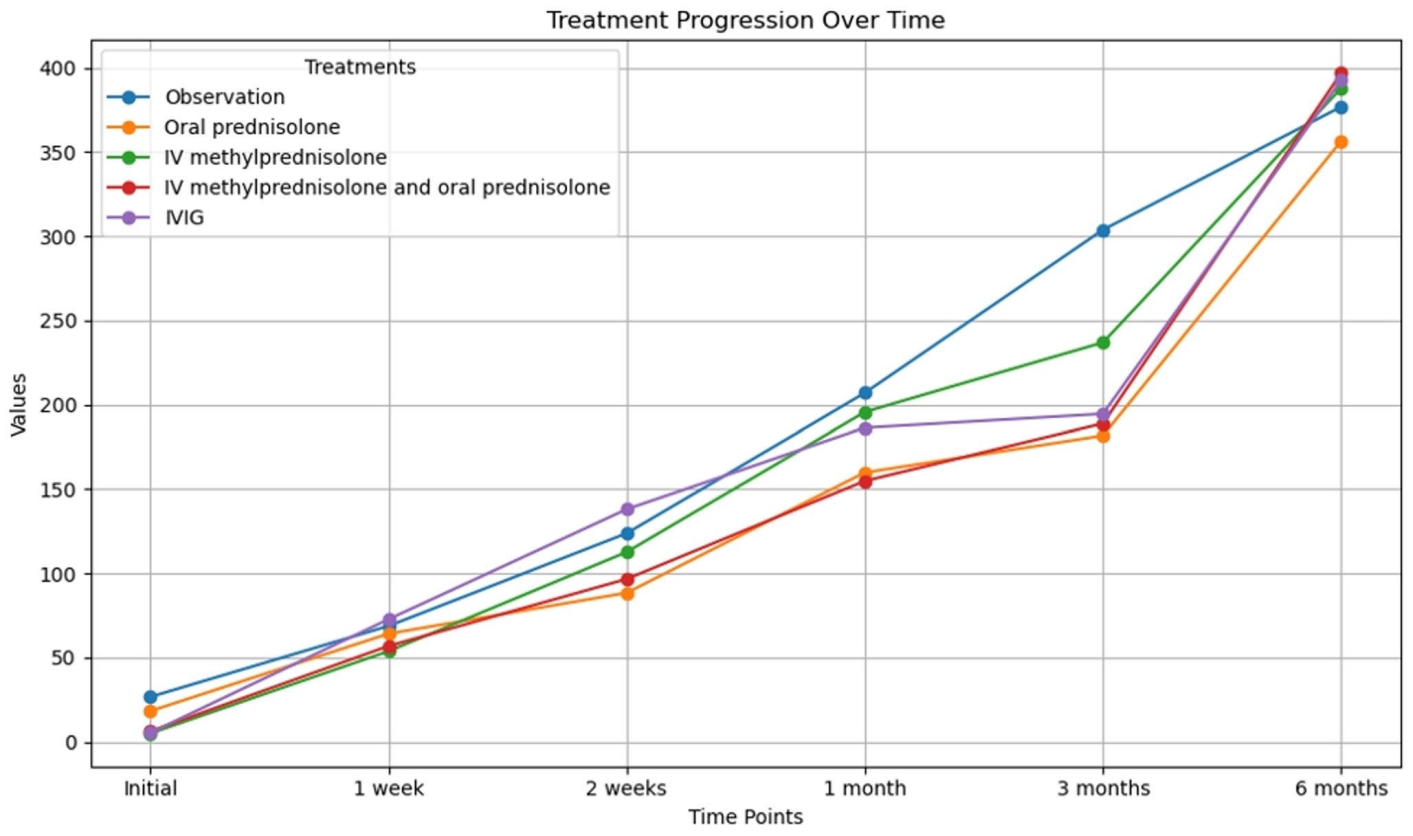
Follow-Up of Platelet Count Among Different First-Line Treatments

**Fig. 2:**
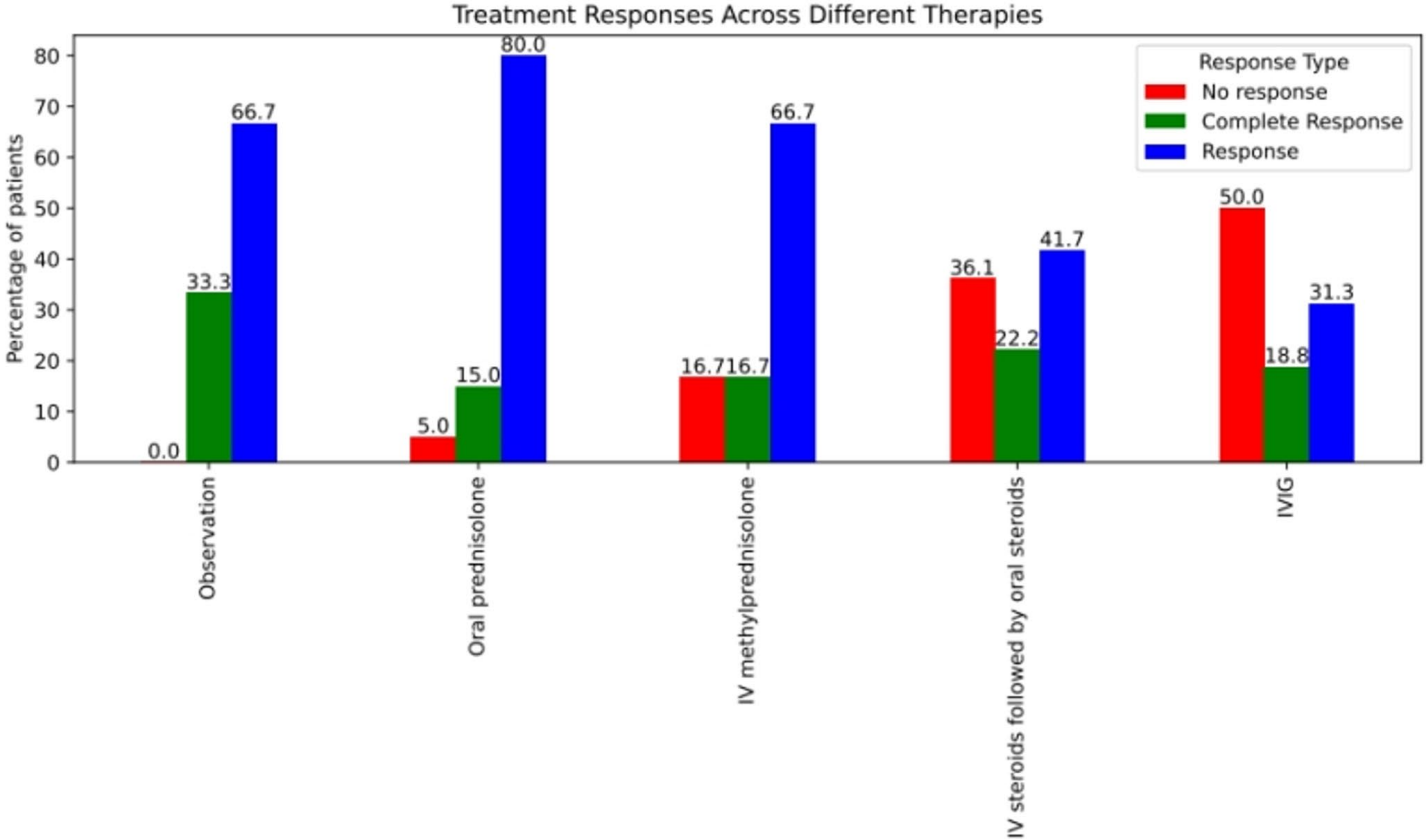
Response After One Week of Different First-Line Treatments

**Fig. 3:**
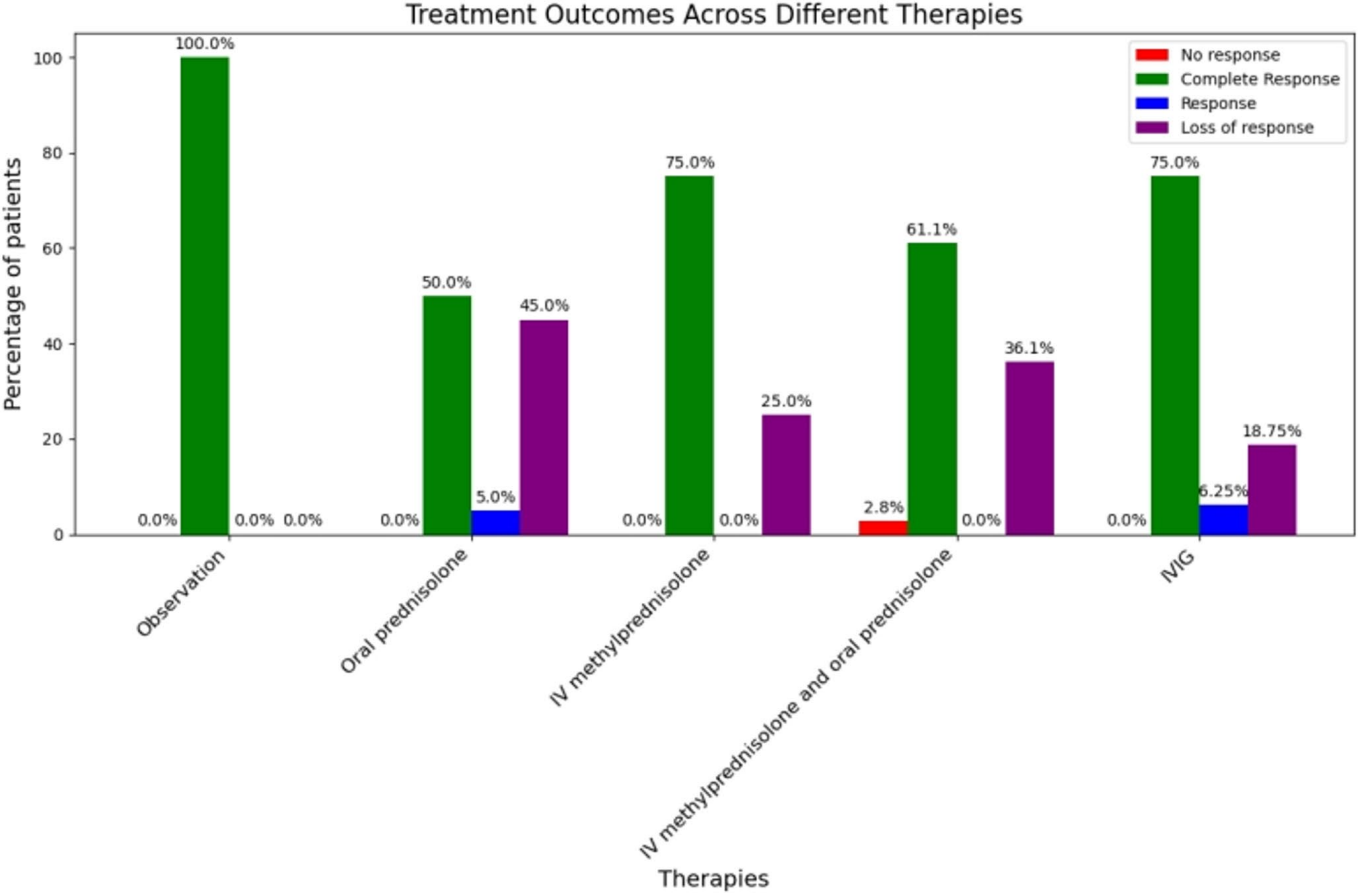
Response After Three Months of Different First-Line Treatments

Laboratory follow-up revealed a marked improvement in the mean platelet count among the study cohort over time. The mean platelet count increased from 9.9 ± 11.9 × 10⁹/L at presentation to 202.4 ± 136.4 × 10⁹/L after three months of treatment, as illustrated in Fig. 1. Figures 2 and 3 depict the treatment responses to various first-line therapies at different time points throughout the study.

**Fig. 4:**
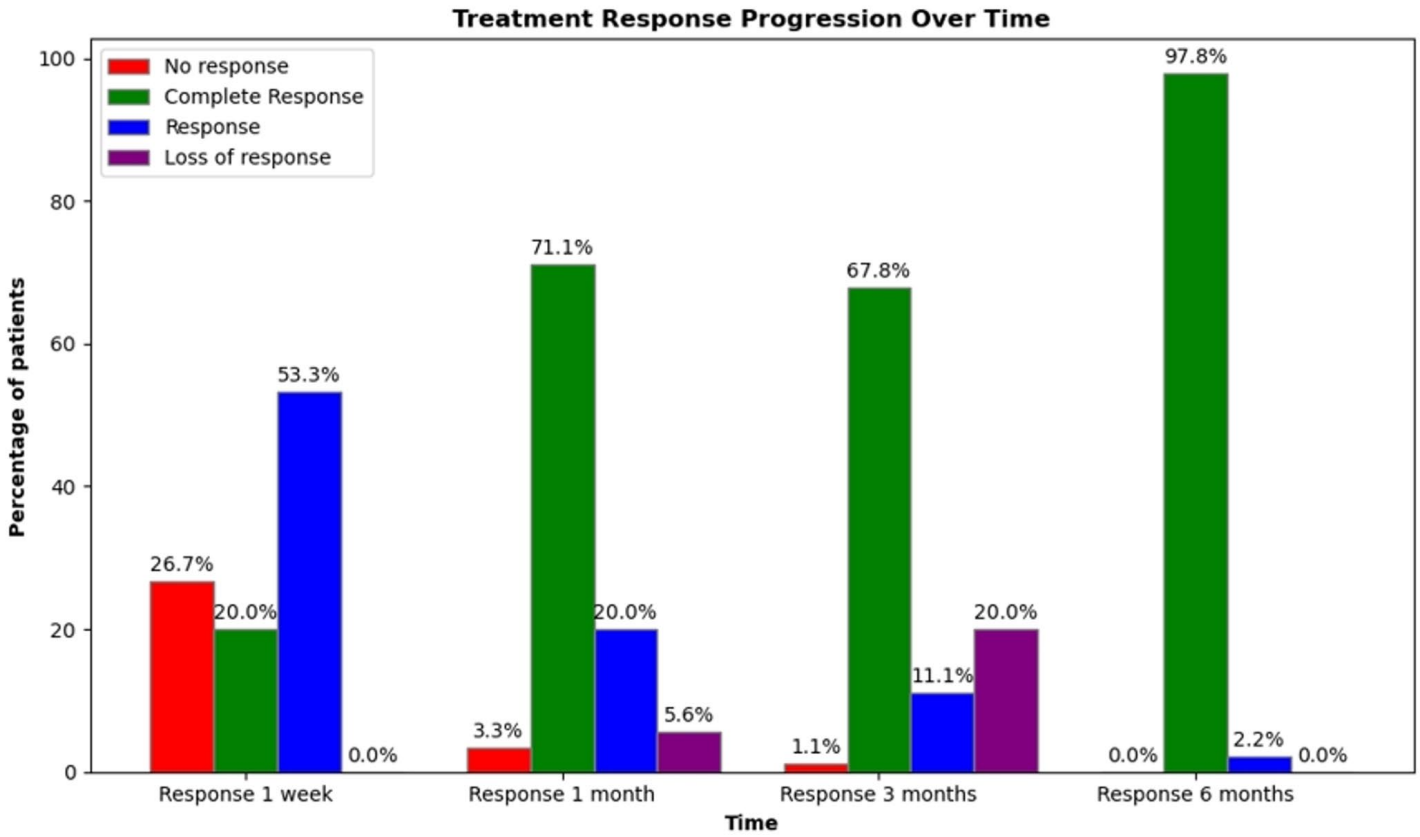
Treatment Response to First-Line Therapy in the Study Cohort

After 3 months of initial treatment, 59 patients (67.8%) achieved CR, 2 patients (11.1%) showed R, 29 patients (32.2%) either had no response (NR) or experienced loss of response (Fig.  4). These 29 patients required second-line treatment with TPO-RAs: eltrombopag was used in 23 patients (79.3%) and romiplostim in 6 patients (20.68%). Among those treated with eltrombopag, 21 patients (91.3%) achieved complete response. All 6 patients (100%) treated with romiplostim achieved complete response as well. The mean platelet count increased from 31.3 ± 5.7 × 10⁹/L before the start of TPO-RA therapy to 279 ± 133.9 × 10⁹/L after three months of treatment.

Comparison of clinical characteristics and treatment lines between responders and non-responders showed that patients who achieved complete response (CR) within six months had a significantly shorter duration from presentation (mean ± SD: 2.9 ± 1.9 days; *p* = 0.003). No other clinical or treatment variables were statistically different between the two cohorts.

Logistic regression analysis for independent factors affecting response showed that the shorter the duration from presentation the better the response (p-value = 0.003), OR (0.769), 95% CI (0.5–0.9).

Among our cohort, two cases remained non-responders (NR) after six months of treatment, despite receiving eltrombopag. The first patient was a 3.5-year-old girl, and the second was a 6-year-old boy. Both had the same BAT score of 2–5 at presentation. The initial platelet count was 2 × 10^9^/L in the first case and 17 × 10^9^/L in the second. Both patients received IV methyl prednisolone followed by oral prednisolone, at presentation. At the six-month follow-up, their BAT scores were less than 2, with platelet counts of 15 × 10^9^/L and 20 × 10^9^/L, respectively.

## Discussion

Immune thrombocytopenia (ITP) is the most common acquired bleeding disorder in children. The primary treatment goals in pediatric ITP focus on improving quality of life and preventing or managing clinical bleeding, rather than solely increasing platelet counts. This observational study aimed to compare the efficacy of various first line and second-line treatment strategies used for newly diagnosed ITP in pediatric patients at Cairo University Children’s Hospital (CUCH).

Upon reviewing the literature, few studies have examined the outcomes of first-line treatment for ITP. Moreover, existing studies often use varying treatment regimens or focus on a single line of treatment without comparing it to alternative approaches.

In our study, while assessing clinical responses to different treatment lines among the study cohort, an interesting pattern emerged. By the end of the first week, a significantly higher proportion of non-responders was observed in the intravenous immunoglobulin (IVIG) group and the group receiving intravenous methylprednisolone followed by oral prednisolone (50% and 36.1%, respectively). In contrast, non-response rates were notably lower with less intensive treatment strategies such as IV methylprednisolone alone, oral prednisolone, or observation (16.7%, 5%, and 0%, respectively), with the difference being statistically significant (*p* = 0.023).

This finding contrasts with the results reported by Haroun et al. [9], who observed significantly higher overall response rates at one week with more intensive treatment regimens. This included pulse IV methylprednisolone followed by oral prednisolone (96.1%), oral prednisolone at 4 mg/kg/day for 4 days (93.3%), and a combination of IVIG with IV methylprednisolone (100%). These were markedly higher than the response rates in groups receiving oral prednisolone at 2 mg/kg/day (58.8%) or no treatment (75%) (*p* = 0.001).

Similar outcomes were noted by Acero-Garcés et al. [10], who found that IVIG (2 g/kg) led to higher response rates at 48 h compared to methylprednisolone (30 mg/kg/day), and at 72 h to 7 days compared to prednisone (2 mg/kg/day).

The discrepancy between our findings and those of previous studies may be attributed to several factors. In the study by Haroun et al., IVIG was administered in combination with IV methylprednisolone, unlike our study, where these therapies were used separately. Furthermore, different dosing protocols were employed across studies. Variability in sample size—both overall and within individual treatment groups—may also have influenced the results. Additionally, in our study, patients receiving less intensive treatment had higher baseline platelet counts, which may have contributed to their better early response rates.

Interestingly, in our study, observation (“wait and see”) was employed as a management strategy for six patients who presented with higher initial platelet counts and no significant bleeding symptoms. All six patients achieved complete response within three months. This finding highlights the potential efficacy of observation without pharmacological intervention in mild cases of thrombocytopenia, reinforcing the notion that some patients may improve spontaneously without the need for treatment. This finding has been similarly reported in previous studies [9] and is supported by recent guidelines for the management of pediatric thrombocytopenia. These guidelines recommend that children and adolescents with ITP who exhibit mild to moderate bleeding (grade 1–3 on the pediatric BAT score) may be managed expectantly, with parental consent, advice, and access to a 24-hour contact point, regardless of platelet count [11].

Importantly, however, the differences in response rates between treatment groups appeared to diminish over time. By the second week and at one month, our study found that the differences in response rates—namely complete response (CR), response (R), and no response (NR)—between treatment groups were no longer statistically significant. This trend is consistent with findings from other studies, such as Haroun et al. [9], who also reported no significant differences in response rates between treatment groups at one and three months.

Our study found a sustained response in 67.8% of patients (responders), defined as maintaining a stable platelet count and absence of bleeding at three months. In contrast, 29 patients (32.2%) required second-line treatment, predominantly due to loss of response (96.6%). Second-line therapies consisted primarily of thrombopoietin receptor agonists (TPO-RAs), including eltrombopag and romiplostim, which demonstrated high efficacy in this refractory or relapsed population. Platelet counts rose significantly from a mean of 30 × 10⁹/L at the time of TPO-RA initiation (around three months) to 280–330 × 10⁹/L by the six-month follow-up. These outcomes are consistent with findings from both clinical trials and real-world studies [12].

Limitations of this study include the relatively small sample size and its single-center design.

Another limitation is that newly diagnosed patients might improve spontaneously irrespective of the line of treatment used or even without treatment so it is hard to say that the treatment improved the outcome, they might have already been recovering because many children do not develop persistent or chronic ITP.

Future research should aim to include multi-center collaborations with a longer follow-up period to better assess long-term outcomes. There is also a clear need for high-quality randomized controlled trials (RCTs) directly comparing specific first-line treatment regimens. Quality of life, cost-effectiveness, and patient-reported outcomes should be the main points of concern.

## Conclusion

Newly diagnosed ITP patients typically respond well to first-line therapies, particularly corticosteroids such as prednisone or methylprednisolone, which continue to serve as the standard initial treatment. Increasingly, treatment decisions are being tailored to the individual patient’s clinical profile. Early identification of non-responders and timely escalation to second-line therapies are essential for optimizing disease control, enhancing patient quality of life, and minimizing treatment-related toxicity.

## Data Availability

No datasets were generated or analysed during the current study.
